# Monomorphic Epitheliotropic Intestinal T-Cell Lymphoma (MEITL) Causing Small Intestinal Perforation Associated With Adnexal Metastasis: A Case Report

**DOI:** 10.7759/cureus.78534

**Published:** 2025-02-05

**Authors:** Kazuya Okamoto, Yutaka Hanaoka, Mika Kuno, Daisuke Kaji, Hironori Uruga

**Affiliations:** 1 Department of Gastroenterological Surgery, Toranomon Hospital, Tokyo, JPN; 2 Department of Hematology, Toranomon Hospital, Tokyo, JPN; 3 Department of Pathology, Toranomon Hospital, Tokyo, JPN; 4 Research, Okinaka Memorial Institute for Medical Research, Tokyo, JPN

**Keywords:** acute care surgery, case report, gastrointestinal perforations, malignant lymphomas, monomorphic epitheliotropic intestinal t-cell lymphoma

## Abstract

Monomorphic epitheliotropic intestinal T-cell lymphoma (MEITL) is a rare and aggressive form of peripheral T-cell lymphoma originating from intestinal epithelial lymphocytes and is associated with a poor prognosis. We present the case of a 50-year-old woman who developed a gastrointestinal perforation, initially suspected to be due to ovarian cancer with peritoneal dissemination. The patient underwent laparoscopic adnexal resection and laparotomy for the intestinal perforation. Postoperative pathology confirmed that the cause of the intestinal perforation and enlarged adnexa was MEITL. Prompt chemotherapy was administered, followed by autologous hematopoietic stem cell transplantation. Despite rapid disease progression, effective management led to favorable treatment outcomes. Early diagnosis and timely intervention are crucial to improve the prognosis of patients with MEITL.

## Introduction

Malignant tumors, particularly those located in the gastrointestinal tract, can cause gastrointestinal perforations. Among the primary tumors of the small intestine, 11.5%-14% of malignant lymphomas lead to intestinal perforations, indicating that malignant lymphomas are at a high risk of intestinal perforation [1]. Monomorphic epitheliotropic intestinal T-cell lymphoma (MEITL), a rare type of lymphoma, frequently causes gastrointestinal perforations. Reports of MEITL complicated by small intestinal perforations are limited [2]. MEITL is an aggressive peripheral T-cell lymphoma originating from intestinal epithelial lymphocytes and is associated with a poor prognosis [3]. MEITL and enteropathy-associated T-cell lymphoma (EATL) account for only 0.25% of all malignant lymphomas and less than 5% of all primary gastrointestinal malignant lymphomas [4]. EATL is associated with celiac disease, with a high incidence in Europe and North America [5]. In contrast, MEITL is not associated with celiac disease and has a higher prevalence in Asia, affecting individuals with a median age of 60.7 years, and 76% of the cases occur in males [6].

Herein, we report a case of MEITL in a 50-year-old woman who presented with a gastrointestinal perforation and had a favorable treatment outcome.

## Case presentation

**Figure 1:**
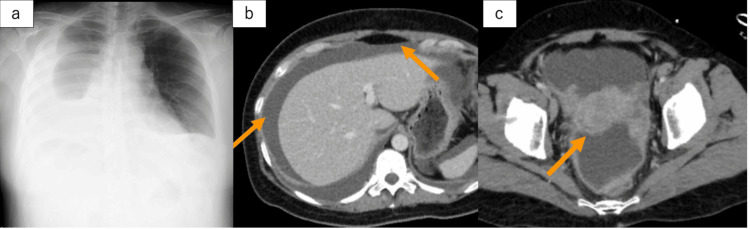
Imaging findings observed at the time of hospital visit (a) Chest radiography showing bilateral pleural effusion with right-sided predominance. (b) Contrast-enhanced CT scan showing ascites and free air in the peritoneal cavity. (c) Contrast-enhanced CT scan showing bilateral adnexal swelling.

A 50-year-old woman presented to the emergency department with chest pain. Her vital signs were as follows: temperature 37.1℃, respiratory rate 20 breaths per minute, SpO₂ 94% on 6 L/min oxygen, blood pressure 130/92 mmHg, and a pulse rate 140 beats per minute. The patient required oxygen support, and her breathing worsened. Physical examination revealed slightly decreased bilateral breath sounds, with no signs of peritoneal irritation. Blood tests showed a hemoglobin level of 17.0 g/dL, white blood cell count of 7,300/µL, C-reactive protein (CRP) level of 1.89 mg/dL, and lactate dehydrogenase (LDH) level of 648 U/L. There was no evidence of worsening anemia or an increased inflammatory response, although elevated LDH levels were noted. Chest radiography showed bilateral pleural effusion with right-sided predominance, and contrast-enhanced computed tomography (CT) revealed ascites, bilateral adnexal swelling, and free air in the peritoneal cavity (Figure 1).

**Figure 2:**
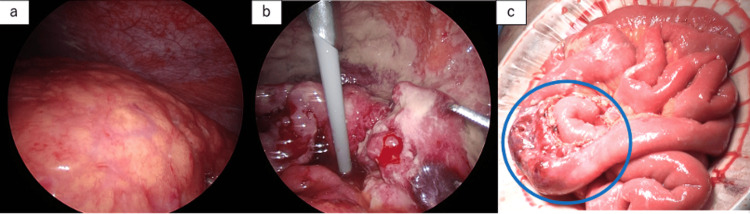
Intraoperative findings (a) Thickened omentum fused into a mass. (b) Enlarged right adnexa. (c) Perforation in the jejunum, 60 cm from the Treitz ligament.

An aspiration kit was inserted into the right pleural cavity to manage the right pleural effusion. Given the clinical presentation, we suspected gastrointestinal perforation secondary to ovarian cancer with peritoneal dissemination and planned emergency surgery. Intraoperative findings revealed stool-colored ascites and a thickened omentum fused into a mass. Laparoscopic right adnexal resection was performed owing to enlarged adnexa. However, because the precise location of the perforation was unclear during laparoscopy, a laparotomy was subsequently performed. This revealed a perforation in the jejunum, 60 cm from the ligament of Treitz (Figure 2).

**Figure 3:**
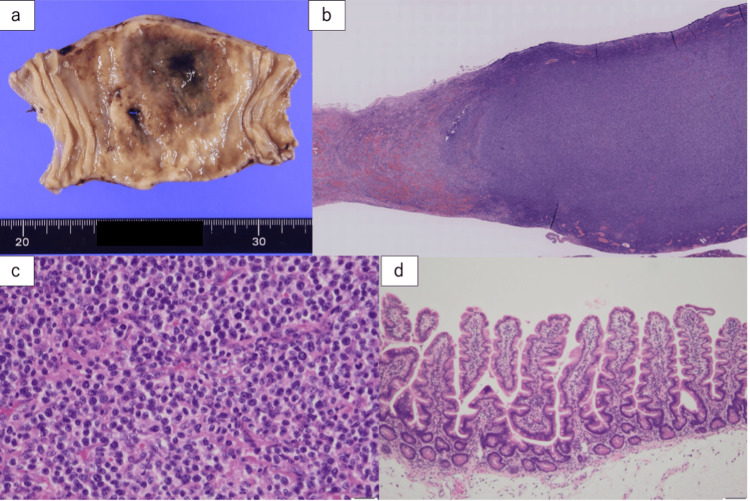
Histopathologic examination of resected small intestine (a) Gross examination showing the small intestine specimen with a whitish appearance, thickened walls, and loss of normal intestinal folds. A 5-mm perforation is present at the center, accompanied by necrotic changes. (b-c) Medium-to-large atypical lymphocytes, with an increasing number of nucleoli, infiltrated the entire layer and necrotic region. (d) Atypical lymphocytes infiltrated the surrounding tissue and lined the epithelium, indicating intraepithelial lymphocytosis.

Partial resection of the small intestine was performed, along with peritoneal lavage and drainage. The operation lasted 3 hours and 8 minutes, with total blood loss, including ascites, amounting to 2,650 mL. Blood transfusions were not required. The intubation tube was removed on the first postoperative day, and oral intake was resumed on the fifth postoperative day. Postoperative blood tests showed an elevated soluble interleukin-2 receptor level of 682 U/mL. Pathological cytological examination of the pleural and ascitic fluids revealed atypical cells with a high nucleus-to-cytoplasm ratio, suggestive of malignant lymphoma. Gross examination of the small intestine specimen revealed a whitish appearance with a thickened wall and loss of normal small intestinal folds. A 5-mm perforation was identified at the center, accompanied by necrotic changes (Figure 3).

**Figure 4:**
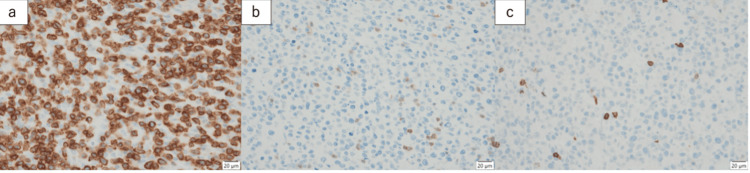
Immunohistochemistry staining supporting the diagnosis of MEITL (a) Lymphoma cells positive for CD3. (b-c) Lymphoma cells negative for CD5 and CD20. MEITL: monomorphic epitheliotropic intestinal T-cell lymphoma.

Histologically, medium-to-large atypical lymphocytes with an increasing number of nucleoli infiltrated the full thickness of the wall and necrotic region. These atypical lymphocytes also infiltrated the surrounding tissues and lined the epithelium, a feature known as intraepithelial lymphocytosis. Immunopathological findings showed that the atypical lymphocytes were positive for CD3, CD8, and CD56 and negative for CD20, CD5, CD4, and CD30 (Figure 4).

**Figure 5:**
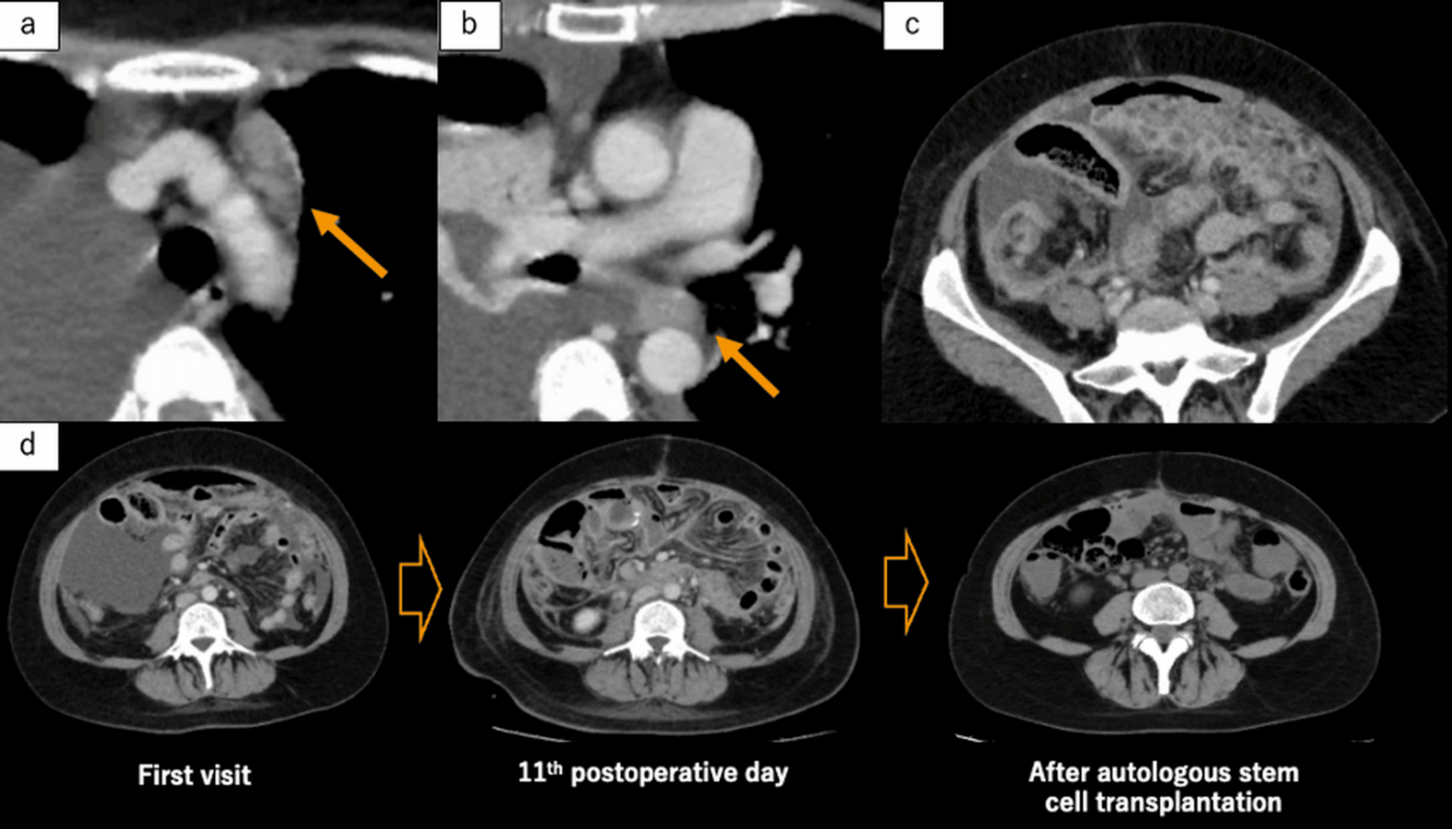
Follow-up contrast-enhanced CT scan on postoperative day 11 (a) Enlargement of the thymic or anterior mediastinal lymph nodes. (b) Enlargement of the lymph nodes below the tracheal carina. (c) Diffuse soft tissue proliferation in the abdominal cavity. (d) The soft tissue proliferation along the mesentery observed on the CT scan at the time of the first emergency department visit worsened on postoperative day 11 but markedly improved following autologous stem cell transplantation.

The Epstein-Barr virus test, performed by in situ hybridization, was negative. Additionally, atypical lymphocytes exhibited diffuse infiltration of the adnexa and omentum. Based on these findings, the tumor was diagnosed as a lymphoma derived from T cells in the intestinal epithelium, and the final diagnosis was MEITL. According to the Lugano staging system, commonly used for gastrointestinal lymphomas, the tumor was classified as stage IV [7]. Furthermore, a contrast-enhanced CT scan taken just 11 days after the surgery aimed at evaluating the disease status before starting chemotherapy revealed rapid disease progression, showing diffuse soft tissue proliferation in the abdominal cavity, enlargement of the thymic or anterior mediastinal lymph nodes, and enlargement of the lymph nodes below the tracheal carina (Figure 5).

Consequently, the patient was promptly referred to the Department of Hematology for chemotherapy. Two weeks after the surgery, the patient began chemotherapy with cyclophosphamide, doxorubicin, and prednisone (CHP) based on the pathological cytological diagnosis of malignant lymphoma. After one cycle of CHP, the patient achieved a partial response, and MEITL was confirmed by postoperative histopathological findings. Consequently, the chemotherapeutic regimen was changed to ifosfamide, carboplatin, and etoposide (ICE). After three cycles of ICE, the patient underwent autologous hematopoietic stem cell transplantation. We are currently closely monitoring the patient’s condition in the outpatient clinic to ensure early detection of any complications or relapse.

## Discussion

Here, we present a case of an aggressive MEITL complicated by intestinal perforation, successfully treated with a comprehensive approach. Preoperatively, the gastrointestinal perforation was suspected to be caused by peritoneal dissemination of ovarian cancer. However, the postoperative pathological diagnosis confirmed that the underlying cause was MEITL. Despite the aggressive progression of the disease, timely intervention enabled the administration of systemic chemotherapy followed by autologous hematopoietic stem cell transplantation.

MEITL is a rare and aggressive form of intestinal T-cell lymphoma with a poor prognosis. The reported one-year survival rate is 38.7%, and the five-year survival rate is 19.7% [8]. This tumor arises from the intestinal intraepithelial T lymphocytes in the intestine and most commonly affects the proximal jejunum of the small intestine [9]. This differentiates it from other primary gastrointestinal lymphomas, which are typically found in the terminal ileum [10]. In the present case, tumor perforation was observed in the jejunum, 60 cm from the ligament of Treitz.

Adnexal metastasis of MEITL, as observed in this case, is extremely rare. While metastasis to the mesenteric lymph nodes is common, metastasis to the pancreas, bladder, brain, and bone marrow has been documented; however, adnexal involvement has not been previously reported [11].

As in the present case, the diagnosis of MEITL can be challenging. Initial symptoms, such as abdominal pain, diarrhea, and weight loss, are often nonspecific, which can lead to misdiagnosis and initiation of treatment for conditions such as enteritis [5,12]. In this case, the patient’s primary complaint was dyspnea, and despite small intestinal perforation, her abdominal pain was mild, with no signs of peritoneal irritation. Furthermore, CT imaging revealed pleural effusion, ascites, and adnexal swelling, which initially raised the suspicion of ovarian cancer with peritoneal dissemination as the cause of gastrointestinal perforation. When lymphoma invades the mesentery and omentum, it often causes tissue thickening and infiltration of cells between adipocytes and collagen fibers [11]. In this case, the CT scan revealed soft tissue proliferation within the mesentery, which provided crucial clues for the correct diagnosis.

Malignant tumors cause gastrointestinal perforations. Among the primary tumors that lead to small intestinal perforation, 11.5%-14% are malignant lymphomas, indicating that these lymphomas are associated with a high risk of intestinal perforation [1]. MEITL is particularly prone to gastrointestinal perforation owing to its pathological characteristics. Atypical lymphocytes infiltrate the entire thickness of the intestinal wall in a destructive manner, without proliferating the connective tissue [13]. Similar histological findings were observed in this case. The tumor itself is fragile, and perforations have been reported even during chemotherapy [14]. As a result, it is essential to closely monitor abdominal symptoms both during and after chemotherapy to promptly detect any changes.

The cases of MEITL with intestinal perforation often follow a rapid postoperative course due to multiple factors, including the patient’s declining general condition, which can postpone the start of chemotherapy, the tumor’s inherent resistance to chemotherapy, and the histopathological fragility of the intestinal tissue [14]. Kawai et al. reported that the median survival for cases of small intestinal perforation is even lower, at 4 months (3 days-75 months) [15]. In this case, the early follow-up CT performed on postoperative day 11 enabled visualization of disease progression at an unusually early stage, revealing significant soft tissue proliferation, peritoneal lymphomatosis, and new lymph node enlargement. Therefore, timely diagnosis and intervention are critical. Bissessur et al. reported that patients who underwent surgical resection followed by chemotherapy and/or autologous stem cell transplantation had better survival rates than those who underwent surgery alone [16]. Therefore, we initiated postoperative chemotherapy just two weeks after the operation. Additionally, we administered ICE chemotherapy (ifosfamide, carboplatin, and etoposide), as this regimen, followed by autologous stem cell transplantation, has been shown to be more effective than anthracycline-based chemotherapy in combination with autologous stem cell transplantation [17].

It is also important to monitor the patient’s condition in the outpatient clinic to ensure early detection of any complications or relapse. The reported progression-free survival is 3.4 months, and early relapse should be considered [17]. No established evidence shows that regular CT follow-up improves prognosis in malignant lymphoma [18]. Likewise, there is an absence of evidence for MEITL. However, routine follow-up imaging might aid in detecting recurrent lesions and potentially preventing intestinal re-perforation. We believe that CT follow-up every 6-12 months is essential for monitoring disease progression.

## Conclusions

This case underscores the importance of considering MEITL as a potential cause of gastrointestinal perforation. Early recognition and prompt intervention with chemotherapy and autologous hematopoietic stem cell transplantation may improve the prognosis of rapidly progressive and aggressive lymphoma. The tendency of MEITL to cause intestinal perforation, combined with its poor prognosis, highlights the need for timely diagnosis, treatment, and careful monitoring of the recurrence to optimize survival outcomes.
